# Dataset on flank wear, cutting force and cutting temperature assessment of austenitic stainless steel AISI316 under dry, wet and cryogenic during face milling operation

**DOI:** 10.1016/j.dib.2019.104389

**Published:** 2019-08-26

**Authors:** Karthik M.C. Rao, Rashmi Laxmikant Malghan, Mervin A. Herbert, Shrikantha S. Rao

**Affiliations:** Department of Mechanical Engineering, National Institute of Technology, Karnataka, Surathkal, 575025, India

**Keywords:** Cooling, Cryogenic, Face milling, Dry, Wet, Tool wear

## Abstract

The contemporaneous examination focuses on the impact of spindle speed as well as an eco-pleasing cooling strategy in the midst of processing of AISI316. Investigations were executed at three different machining approaches viz. dry, wet and cryogenic (LN_2_) using cemented carbide inserts. Water dissolvable oil was used as a cutting fluid for flood cooling approach. The workpiece was processed under three distinctive cutting speeds i.e. 1000, 2000 and 3000 rpm however feed rate and depth of cut were kept consistent at 450 mm/min and 1mm separately. An exhaustive investigation on the cooling impacts of LN_2_ technique on a segment of the significant machinability perspective, for instance, cutting force (Fx), insert wear, surface quality and processing temperature is delineated. The beforehand specified processing responses were documented and contrasted all together to demonstrate the reasonableness and achievability of LN_2_ approach in examination with dry and wet machining methodology. The outcomes accomplished in the midst of the examination obviously settled the commonness of realizing LN_2_ for achieving upgraded machinability inside a predetermined scope of process parameters.

**Table:** Specifications Table

Subject	Cryogenic (LN_2_)
Specific subject area	Cryogenic/Face milling operation- Tool wear
Type of data	Table (Experimental conditions) Graph (Cutting force, Cutting temperature) Figure (Chip morphology, Optical microscopic images of flank wear)
How data were acquired	Data was acquired by experimental techniques - CNC vertical milling machine (Experimental data collection-machining- dry,wet, cryogenic conditions) - Indirect method-ethernet cable -FANUC-servo guide s/w (cutting force) - Scanning electron microscope (Tool wear, Chip morphology) - Infrared thermometer - Fluke 59 mini (Cutting temperature)
Data format	Cutting temperature, Flank wear, Chip morphology - Raw Cutting force- Calculated
Parameters for data collection	Face Milling – AISI316 machining Experimental data were collected under various machining conditions: Dry, wet and cryogenic (LN_2_) Input Variables: Spindle speed(rpm), Feed rate(mm/min) and Depth of cut (mm) Output Variables: Cutting temperature, cutting force, flank wear, chip morphology.
Description of data collection	Milling process of AISI 316 under different machining environments (Dry, wet and cryogenic (LN_2_) conditions) Variation of Cutting temperature, Cutting force, Flank wear at various spindle Speed of 1000,2000 and 3000 rpm with constant feed rate of 450mm/min and depth of cut of 1mm.
Data source location	Surathkal, India;
Data accessibility	Direct URL to data: [ https://link.springer.com/article/10.1007/s12666-018-1473-y]
Related research article	Author's name: MC Karthik Rao, Rashmi L Malghan, S ArunKumar, Shrikantha S Rao, Mervin A Herbert Title: An Efficient Approach to Optimize Wear Behavior of Cryogenic Milling Process of SS316 Using Regression Analysis and Particle Swarm Techniques Journal: Transactions of the Indian Institute of Metals DOI: https://link.springer.com/article/10.1007/s12666-018-1473-y Author's name: Karthik Rao, Arun Kumar Shettigar, Rashmi L Malghan, Shrikantha S Rao, Mervin A Herbert Title: Machinability Study of Austenitic Stainless Steel under Wet and Cryogenic Treatment in Face Milling Journal: Journal of Materials Science & Surface Engineering DOI: http://www.jmsse.in/files/563_Karthik_Rao_et_al.PDF

**Table:** 

**Value of data** • Data can be used to explain the behavior and effects of fluids on AISI316 in milling process. • This dataset can be used to compare the effect of dry, wet and cryogenic responses with fluids in other machining process. • The experimental data suggest the impact of fluids in reducing the temperature in tool-workpiece interface and achieving better surface finish in machining. • Our data could inspire others to investigate on different materials using cryogenic machining and to carry out the comparative investigation using various fluids.

## 1 Data

Data presented in the article is pertaining to AISI 316 stainless steel behavior under different machining environments. The experiments were performed to evaluate the behavior and effects of fluids on AISI316 in face milling process. Table 1 indicates the experimental conditions utilized to carry out the machining environments with various process parameters such spindle speed of 1000–3000 rpm, feed rate of 450 mm/min and depth of cut of 1mm. The Output Variables such as Cutting temperature, flank wear, chip morphology is experimentally investigated (Raw) and Cutting force experimentally investigated (Calculated-using indirect method -ethernet cable -FANUC-servo guide software). Later the data has been compared inorder to investigate the effect of responses under different considered environments.

**Table 1:** Experimental conditions.

Workpiece Material & Size	AISI 316 (110 mm × 38 mm × 25mm)
Face Milling Process Parameters	Spindle Speed: 1000,2000,3000 rpm
	Feed Rate: 450 mm/min,
	Depth of Cut: 1mm
Environments	Dry,
	Wet,
	Cryogenic (LN_2_).

## 2 Experimental design, materials and methods

### 2.1 Test equipment

**Fig. 1:**
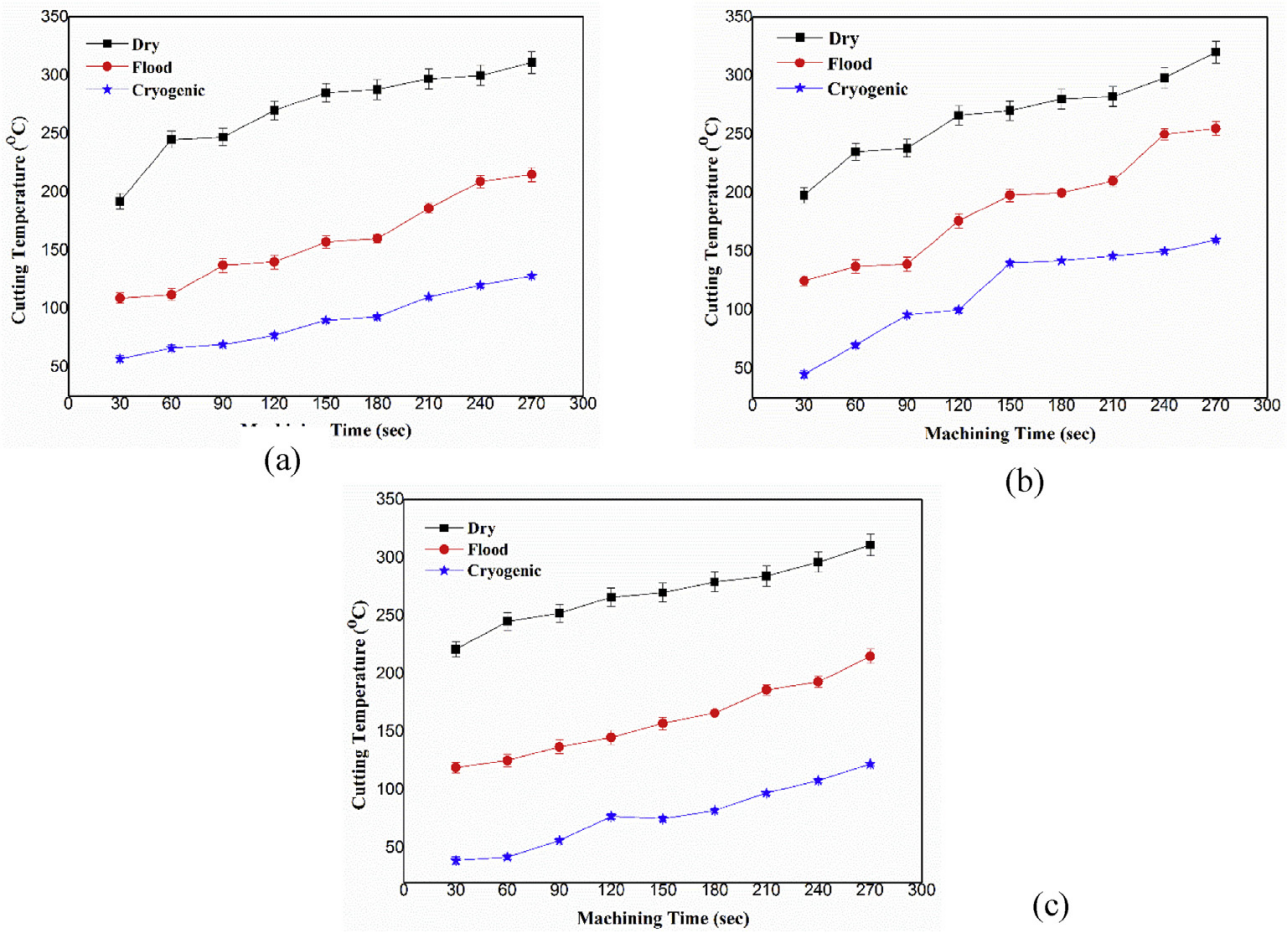
Variation of Cutting Temperature at speed of (a) 1000 rpm (b) 2000 rpm (c) 3000 rpm.

Table 1 represents the investigational conditions utilized in the present work. CNC Spark DTC-12 was used to complete processing investigates AISI 316 stainless steel. LN_2_ cryogenic experimental arrangement is described in Fig. 1. Trial experiments were completed for 1000, 2000 and 3000 rpm. Constant feed rate and depth of cut of 450 mm/min, 1 mm respectively. The limit furthest reaches of parameters relied upon the preparatory investigations directed.

### 2.2 Method: cryogenic cooling method

An insignificant cost setup of cryogenic cooling was used to infringe the LN_2_ at the tool workpiece interface. The setup incorporates TA55 cryocan, it is used to stock up the LN_2_ by means of the air compressor. Flow of pressurized LN_2_ is given through the specially designed nozzle to the tool-workpiece interface with the pressure of 3 bars and rate of flow of LN_2_ is 0.33 l/min.

### 2.3 Measurement of performance characteristics

The surface roughness of each LN_2_ machined surface was measured utilizing the Mitutoyo surface roughness analyzer [1], [2]. Three readings at various areas were measured on each machined surface and the normal of it was considered as the definitive estimation of surface roughness estimation [3], [4], [6]. Infrared thermometer was used to evaluate the cutting temperatures at the instrument workpiece interface.

#### 2.3.1 Cutting temperature (CT) variation in different evironments

Cooling strategies are employed by an objective to diminish the CT [7]. These are likewise help-full in keeping the cutting tools from extreme impairment like adhesion, diffusion and abrasion which are emphatically co-identified with machining temperature. In addition, at machining AISI316 at raised temperature prompts to swift device wear. The disparity attained in CT amid examination is appeared in Fig. 1. It's clear through Fig. 1 that, as the spindle rotational speed increases, the processing heat increases linearly at machining sector because of enhanced friction at device work interface. In case of LN_2_, the machining temperature was relatively cut down for considered scope of spindle speeds in contrast to dry and flood strategies. Remarkable diminishments in processing heat were seen under LN_2_ circumstance than that of other strategies (dry, flood). This is on the grounds that, the heat exchange happens amid LN_2_ processing approach over convection and dissipation. Utilization of LN_2_ superbly encourages the liquid beads to reach at device workpiece interface which offer a proficient warmth exchange in this manner gives enhanced lubrication prompting to low CT. Hence it may be added to prevalence of coolant particles to enter efficaciously in to the cutting zone.

#### 2.3.2 Cutting force (Fx) variation in different evironments

**Fig. 2:**
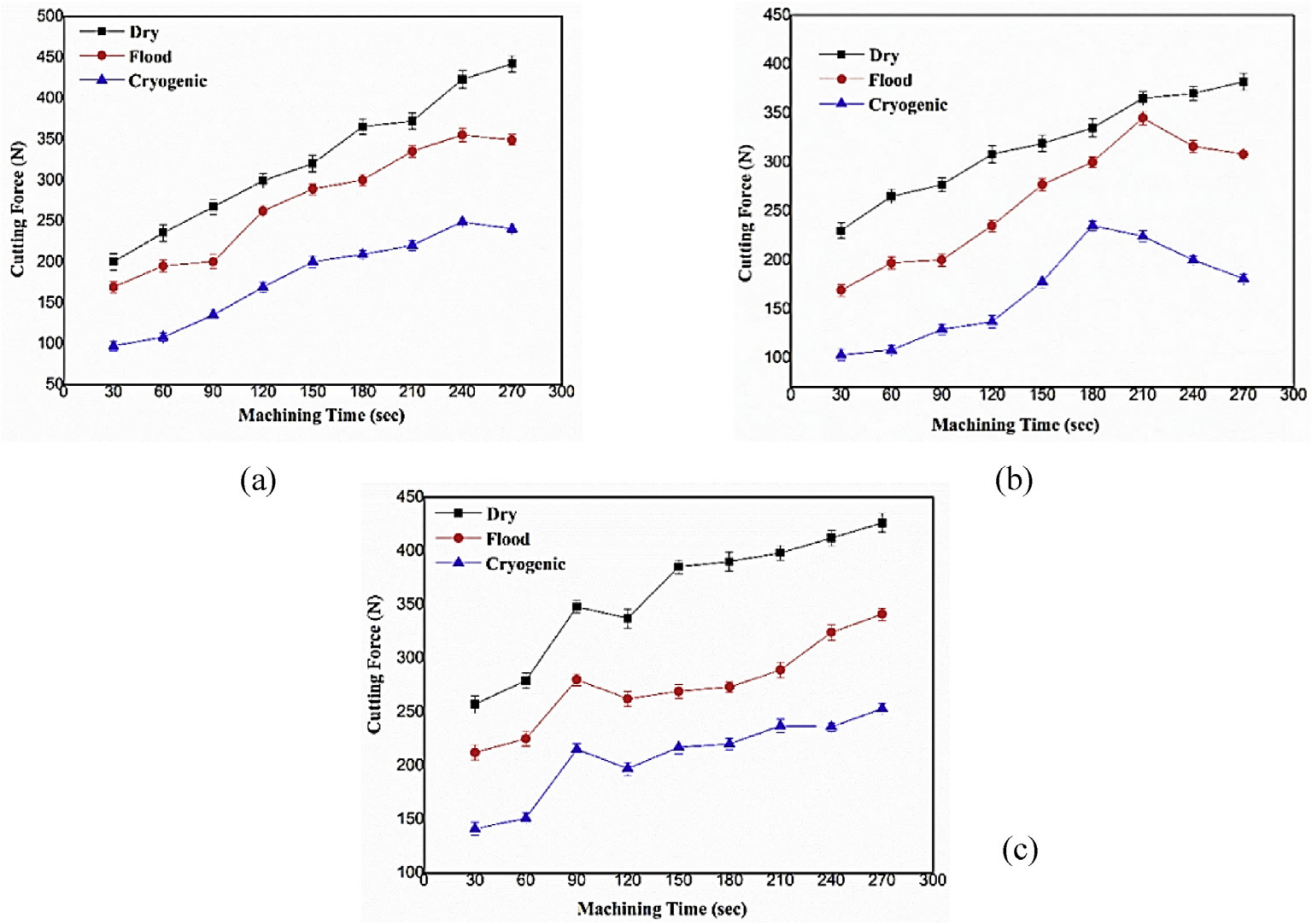
Variation of Cutting Force at speed of (a) 1000 rpm (b) 2000 rpm (c) 3000 rpm.

The investigation explores divergence of Fx in dissimilar machining conditions since it show a relationships with the further crucial cut attributes, for example, tool wear, processing temperature at cutting zone, surface finish [1], [5]. Fig. 2 depicts the variety in Fx response concerning processing span (time) under dry, with wet and LN_2_ strategy for face milling. Fig. 2 demonstrates the Fx response which shows an expanding pattern with processing condition. It adds to warm mollifying of the workpiece at raised heat condition and spindle rotational speed. Rapid consistent processing in like way contributes in cutting down the shear quality of the workpiece and subsequently decreases the Fx. Captivatingly, LN_2_ method of machining brought exceptional diminish in cutting force intensity to 43% and 16% in examination with dry, flood circumstances exclusively [7], [8], [9]. It is evident from Fig. 2, that cryogenic strategy helps in diminishment of Fx hence, attainable inferences like reduced tool wear and better external finish is achieved. Likewise, noteworthy variation in Fx is attained via flood and LN_2_ mode for speed range of 1000–3000 rpm.

#### 2.3.3 Investigation of flank Wear(FW) variation in different evironments

Tool wear amid processing of AISI316 was described by flank wear, in this way contemplated with the assistance of SEM. Table 2, demonstrates the movement of average FW for various processing span at the spindle speed of 1000, 2000, 3000 rpm via dry, flood and LN_2_ cutting strategies. A quick development in FW seen after 60 seconds machining at spindle speed (2000–3000 rpm), particularly while machining under dry cutting condition. Then again, no huge variation was noticed at first up to 60s for all the examined scope of spindle speeds in all machining condition. However, the prevalence of using LN_2_ was a prominently obvious for greater spindle speed as it enhances cooling in contrast to dry and flood strategies, thus prompts to lower FW. Along these lines LN_2_ emerged as a reasonable option for machining AISI316.

**Table 2:** Optical Microscopic Images Depicting progression of Flank Wear.

Input Variables	Machining Type	Time in seconds		
		30	60	90
Spindle speed =1000rpm Feed rate =450 mm/min Depth of cut = 1mm	Dry			
	Wet			
	LN_2_			
Spindle speed =2000rpm Feed rate =450 mm/min Depth of cut = 1mm	Dry			
	Wet			
	LN_2_			
Spindle speed =3000rpm Feed rate =450 mm/min Depth of cut = 1mm	Dry			
	Wet			
	LN_2_			

Table 2, exhibits the microscopic pictures of flank wear alongside machining span under all machining conditions. High substance reactivity of AISI316 prompts adhesion of the material to tool confront which brings about the creation of built up edge (BUE) [10]. The higher friction was observed in machining span of 60 seconds under strategies (dry, flood) in contrast to LN_2_. Table 2 likewise shows inclination of BUE generation was more conspicuous amid dry mode when contrasted with wet mode. Tremendous cooling in mix with great lubrication brought about critical decrease in friction and along these lines added to the remarkable cutting inserts performance especially under LN_2_ mode even at higher spindle speed.

#### 2.3.4 Investigation of chip morphology (CM) variation in different evironments

**Fig. 3:**
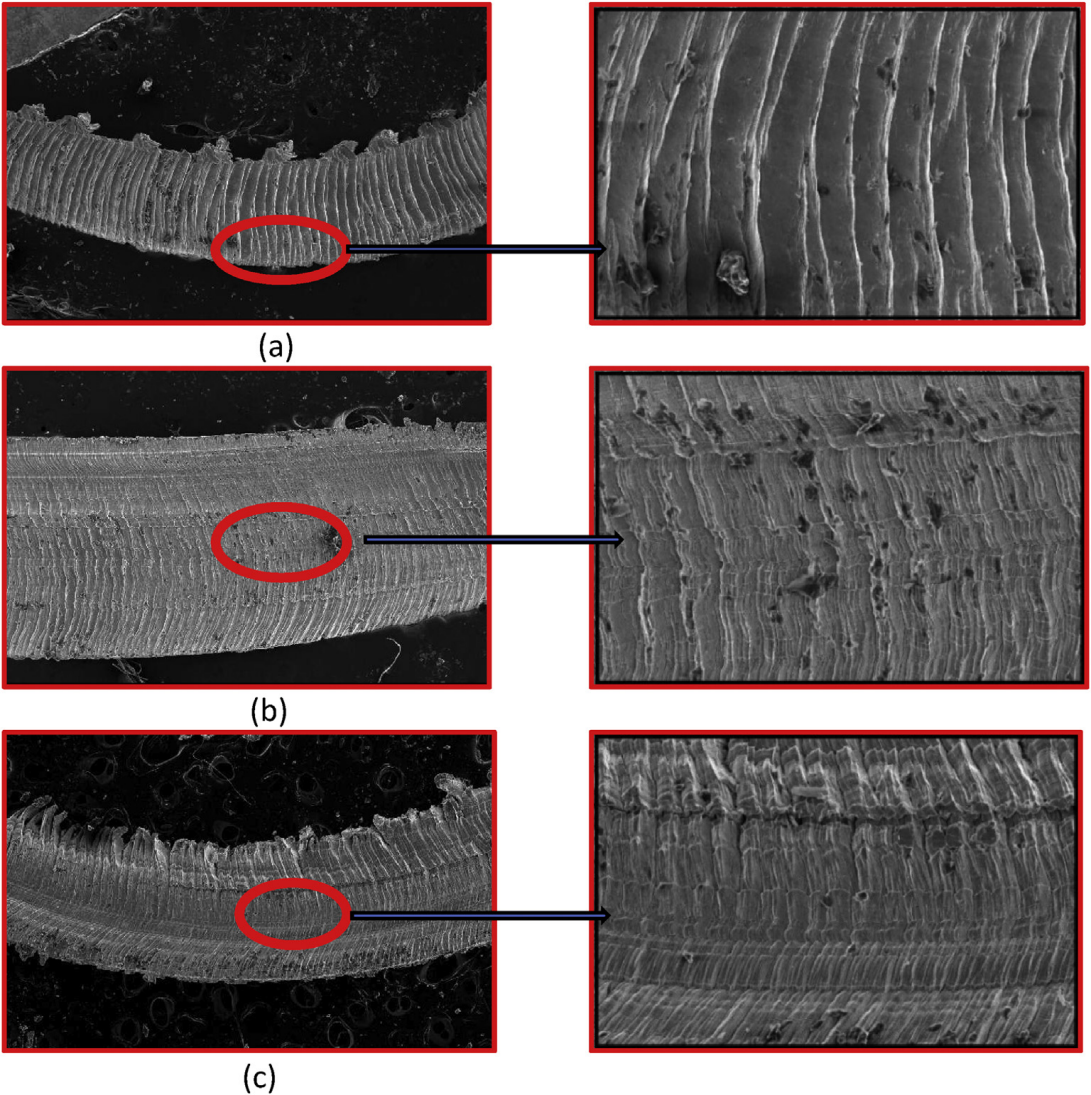
SEM images of chips produced under (a)dry (b)flood (c) Cryogenic conditions.

The CM is delivered in the milling of the workpiece was profoundly analysed with the assistance of the SEM pictures as portrayed in Fig. 3. Generally, AISI316 is described by notched chips as confirmed in Fig. 3. It is fundamentally credited to shear localisation and plastic distortion of the workpiece amid processing. In addition, processing AISI316 at elevated spindle rotational speed (3000 rpm) leads to high rate of friction at contact asperities in turn leading to higher rate of plastic deformation thus creates more notched chips via dry strategy. Along these lines, level of serration was seen to be more under dry condition when contrasted with that of wet machining condition. It may be credited to greater CT development at cutting precinct in dry strategy. Thus, high CT prompts a noteworthy upgrade in the plasticity of the machined samples [8], [9]. At the other cases of machining, the distortion of the machined samples via flood and LN_2_ strategy was less because of lower CT. This may be due to the side stream of chip material, as apparent through previously mentioned Fig. 3.
