# Supplementary material for: Dataset on flank wear, cutting force and cutting temperature assessment of austenitic stainless steel AISI316 under dry, wet and cryogenic during face milling operation
Source: Data Brief. 2019 Aug 26;26:104389. doi: 10.1016/j.dib.2019.104389 (PMC6737182; doi:10.1016/j.dib.2019.104389)
Supplement: Multimedia component 1 [file mmc1.pdf]

Trans Indian Inst Met (2019) 72(1):191–204
https://doi.org/10.1007/s12666-018-1473-y

TECHNICAL PAPER

An Efficient Approach to Optimize Wear Behavior of Cryogenic Milling Process of SS316 Using Regression Analysis and Particle Swarm Techniques

M. C. Karthik Rao[1] · Rashmi L. Malghan[2] · S. ArunKumar[3] · Shrikantha S. Rao[1] · Mervin A. Herbert[1]

Received: 2 June 2018 / Accepted: 5 November 2018 / Published online: 13 December 2018
© The Indian Institute of Metals - IIM 2018

Abstract The present work is an endeavor to carry out a machining using LN_2 in face milling operations and to produce the milling samples with excellent wear resistance property. The output response (wear rate) depends on appropriate choice of speed, feed, and depth of cut. The experimental data are conducted (collected) for SS316 as per central composite design. The present work exemplifies an employment of conventional and nonconventional strategies for optimizing the milling factors of cryogenically treated samples in face milling to achieve the desired wear (response). The results of nonlinear regression (desirability strategy) and nonconventional [particle swarm optimization, (PSO)] optimization techniques are compared, and PSO is found to outperform the desirability function approach. The present work even highlights the effect and results of LN_2 on wear in contrast to wet condition.

Keywords Cryogenic · Optimization · Conventional · Nonconventional · Milling · Central composite design · Desirability · Particle swarm optimization · Wear

✉ Rashmi L. Malghan
rashmi.malghan@gmail.com

M. C. Karthik Rao
karthikmvit@gmail.com

S. ArunKumar
ak.shettigar@manipal.edu

Shrikantha S. Rao
ssrcsr@gmail.com

Mervin A. Herbert
merhertoma@gmail.com

[1] Department of Mechanical Engineering, NITK, Surathkal 575025, India

[2] Department of Computer Science Engineering, Madanapalle Institue of Technology and Science, Madanapalle 517325, India

[3] Department of Mechatronics Engineering, Manipal Institute of Technology, Manipal Academy of Higher Education, Manipal 576104, India

1 Introduction

Austenitic stainless steel is a standout among the most engineering materials with a wide assortment of utilization. Better resistance to erosion and similarity in high temperature and a high vacuum has especially settled on it an appealing decision. SS316 is generally used in marine exteriors, chemical processing equipment, and food processing equipment. Stainless steel is otherwise called corrosion-resistant steel since it is an iron-based steel combination, which contains at least 11% chromium. Chromium present in it keeps it from getting corroded. At the point when conventional carbon steel is presented to rainwater, it erodes effectively because of the development of a dark-colored iron oxide at a first glance, which is regularly called rust. In any case, when more than around 10% chromium is added to standard steel, the oxide at a first glance changes. Stainless steel, for most of the part, has high malleability and weldability properties.

Machining industries are utilizing ordinary liquid coolants at the metal cutting area to defeat the increasing temperature [1]. Nonetheless, it has been discovered that flood cooling strategy does not diminish the cutting temperature at device chip interface at higher cutting liquids and regular cutting liquids is concoction of contaminants which cause a few health and environmental issues and

extra transfer cost (Shaw et al. [2], Cassin et al. [3] and Baradie [4]). Likewise, it is determined that the related costs identified with cutting liquids are 30% of aggregate assembling costs Pusavec et al. [5].

A few of ecologically cognizant controls constrain the enterprises as far as utilization and transfer of customary slicing liquids is concerned as a result of natural contamination affect (Hong et al. [6]). In this way, there is a need to focus on green assembling to defeat natural contamination, cutting liquid transfer, support, upkeep, and lower profitability issues. Machining with a usage of liquid nitrogen (LN_2) is one of the considerate strategies. Subsequently, in the current work, a cryogenic splash cooling system is utilized, i.e., streaming of the LN_2 (196 °C) at the tool–workpiece periphery. LN_2 generously decreases the temperatures at the tool–chip periphery and creates lesser coefficient of friction (Hong et al. [7]). Bordin et al. [8] indicated that the usage of LN_2 lowers tool wear and good surface finish is achieved in contrast to dry machining amid machining of titanium amalgam. Jerold et al. [9] demonstrated that CO_2 yields higher instrument wear in contrast to the wet machining for AISI 1045 steel. Umbrello [10, 11] illustrated better surface finish in cryogenic machining in contrast to the dry machining amid machining of AISI 52100 steel. Klocke et al. [12] observed lower tool wear and reduced surface roughness in a cryogenic condition in contrast to minimum quantity lubrication (MQL) machining conditions because of a considerable decrease in the cutting temperatures. Thus, cryogenic machining expands the efficiency and quality of the item in the machining of gamma titanium aluminides in contrast to other wet, MQL machining. Tandon et al. [13] implemented particle swarm optimization to optimize multifactors, and results indicate saving of machining time by 35%. Basker et al. [14] incorporated numerous nonconventional strategies (genetic algorithm, tabu search, ant colony algorithm, and particle swarm optimization) for optimizing machining parameters in milling operation. Mukherjee et al. [15] proposed an optimization approach to identify the optimal cutting conditions or optimal conditions in several categories of metal cutting process. Raja et al. [16] generated a strategy to predict the surface finish in milling for aluminum material by adopting the PSO technique. Julie et al. [17] implemented Taguchi approach to optimize the surface roughness in process of milling. The authors analyzed the experiments using analysis of variance (ANOVA) and concluded that the Taguchi approach is successful in optimizing the surface quality.

To the author's knowledge, very less report is accessible on machining of AISI 316 via cryogenic condition. From the literature, it can be perceived that by the use of LN_2, the life of the tool rises and attains better surface quality amid machining of material which is hard to cut; regardless of many points of interest, it over cools the surface, and the machined surface hardness also increases. Thus, the point of the recent research is to consider the impact of LN_2 as the cutting fluid on cutting temperature and surface integrity in the processing of AISI 316 over the wet machining. In the current work, an effort is made to incorporate the optimization strategy to optimize the responses using PSO, as very limited amount of work has been performed to anticipate the response in milling operation.

2 Experiment, Materials, and Methods

2.1 Experimental Procedure and Optimization

To achieve the stated goals of the current work, four phases have been followed and these are conferred below:

2.1.1 Step 1: Selecting Milling Factors and Respective Levels

The decision of machining factors and choice on the working levels are of central significance to persuade over the procedure and abate the imperfections. In order to decide the work levels at the initial stage, the preliminary experiments have been conducted by considering the machine capacity, type of tool, material, and coolant. Based on the attained results, levels of the parameters have been selected. The cutting tool used to carry out the experiments is SDMT 1204PDR-MH-TN450. Too wide working scope of factors leads to infeasible elucidation on the retort surface; on the other hand, excessively limited range will bring about inadequate or poor data about the process (Reddy et al. [19] and Rashmi et al. [20]). Design of experiment and RSM methods are pragmatic to contemplate and evaluate the impression of factors on responses. Accordingly, accessing the available literature, Doyen's recommendation and trial tests directed at the exploration research center are utilized to choose process factors and fix their working range. Table 1 exemplifies the considered input factors and their respective operating ranges.

2.1.2 Step 2: Conduction of Experiments

CCD is a largely acceptable and utilized nonlinear regression strategy to fit the response surface. Experiments are carried out according to CCD design matrices and are represented in Table 2. In the present work, for the CCD model, thirty-one experimental runs are carried out, and these runs deliver the complete perception of point-by-point comprehension of all input terms over the outcomes (Rashmi et al. [18]). Further, fifteen distinctive

Table 1 Cutting parameters and their levels

Machining parameters	Units	Notation	Operating levels		
		Coded	Low (− 1)	Middle (0)	High (+ 1)
Spindle speed	(rpm)	S	1000	2000	3000
Feed rate	(mm/min)	F	350	450	550
Depth of cut	(mm)	D	0.5	1	1.5
Coolant type		CT	− 1(Dry)	0(Wet)	1(LN_2)

Table 2 Experimental design matrices of RSM model for response (wear and surface roughness)

Runs	Spindle speed (rpm)	Feed rate (mm/min)	Depth of cut (mm)	Coolant type
1	− 1	− 1	− 1	− 1
2	1	− 1	− 1	− 1
3	− 1	1	− 1	− 1
4	1	1	− 1	− 1
5	− 1	− 1	1	− 1
6	1	− 1	1	− 1
7	− 1	1	1	− 1
8	1	1	1	− 1
9	− 1	− 1	− 1	1
10	1	− 1	− 1	1
11	− 1	1	− 1	1
12	1	1	− 1	1
13	− 1	− 1	1	1
14	1	− 1	1	1
15	− 1	1	1	1
16	1	1	1	1
17	− 1	0	0	0
18	1	0	0	0
19	0	− 1	0	0
20	0	1	0	0
21	0	0	− 1	0
22	0	0	1	0
23	0	0	0	− 1
24	0	0	0	1
25	0	0	0	0
26	0	0	0	0
27	0	0	0	0
28	0	0	0	0
29	0	0	0	0
30	0	0	0	0
31	0	0	0	0

(experiments) blends of milling variables have been carried out to validate (test cases) models.

2.1.3 Step 3: Development of CCD Model, Statistical Investigation, and Performance Assessment

The data represented in Table 2 are utilized to generate a CCD model. The common type of polynomial function (second order) is signified underneath:

$$\hat{Z}_1 = z - \varepsilon = b_0x_0 + b_1x_1 + b_2x_2 + b_3x_3 + b_4x_4 + b_{11}x_1^2 + b_{22}x_2^2 + b_{33}x_3^2 + b_{44}x_4^2 + b_{12}x_1x_2 + b_{23}x_2x_3 + b_{13}x_1x_3 + b_{14}x_1x_4$$

The previously mentioned response surface function incorporates linear (X_1, X_2, X_3, X_4), quadratic (X_1^2, X_2^2, X_3^2, X_4^2), two-term factor interaction ($X_1{*}X_2$, $X_1{*}X_3$, $X_1{*}X_4$, $X_2{*}X_3$, $X_2{*}X_4$ and $X_3{*}X_4$) and the error term (ε) where z is considered to be a response [log scale $x0 = 1$ (dummy factor)], $x1$, $x2$, and $x3$ are log conversions of spindle speed, feed rate, and depth of cut, respectively, whereas b_0, b_1, b_2, b_3 and b_4 are the factors to be appraised.

The coefficients are determined firstly by means of accumulating milling (control–response) data, and next is to execute the model which is constructed via regression analysis. The collected control and response data are analyzed, and nonlinear control–response associations are established. The fundamental factors and interaction of factor's effect is analyzed. The model is verified for its efficacy and significance by incorporating ANOVA strategy. The software tool (Minitab) has been incorporated for the stated goal. Further, to overview the CCD performance, the ten test cases are involved.

2.1.4 Step 4: Optimization of Milling Process Parameters

No impeccable general prerequisites are characterized yet to distinguish the best set of milling parameters. Currently, there are numerous optimization tools accessible and each individual has distinctive constraint and focal points. The model performance for most of the parts rely upon the specific issue area and process multifaceted nature. The current work compares the performance of DFA and PSO strategies to distinguish milling process variables that will yield preferred responses.

2.2 Material and Experimental Setup

Table 3 depicts the experimental conditions utilized in the current work.

CNC Spark DTC-12 has been utilized to carry out face milling experiments on AISI 316 stainless steel (100 mm × 40 mm × 30). LN_2 machining setup is delineated in Figs. 1, 2, and metal cutting region is represented in Fig. 3. The chemical composition of the material is represented in Table 3.

3 Results and Discussion

In this segment, milling process modeling and optimization are mentioned. The phases and methodology followed to mannequin and optimize the milling for the responses are illustrated in Fig. 4.

The experimental data have been accrued as per outline matrices of CCD. The surface plots and the corresponding input and output relations are generated using the gathered data. Further, using analysis of variance (ANOVA), the statistical sufficiency of the strategy has been evaluated. The accuracy of the prediction of the created model is verified with 10 test cases, and the superlative one is chosen in light of the average absolute percent deviation esteem. The best ideal milling prerequisites accountable for the desired responses are resolved using DFA and PSO (Rashmi et al. [18]). At long last, the best optimization technique is opted by evaluating the performance between conventional (DFA) and nonconventional (PSO).

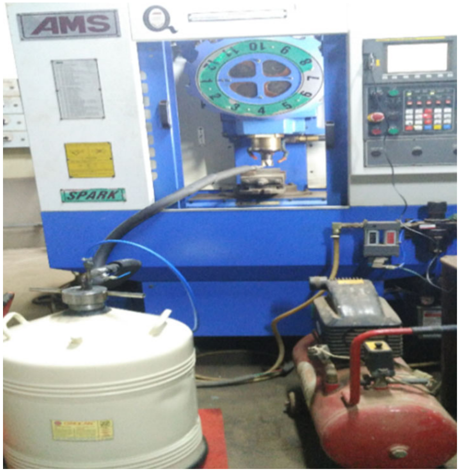

Fig. 2 Cryogenic machining setup

3.1 Development of Model and Statistical Analysis

The experimental accumulated control–response data are applied for the generation of mathematical (nonlinear) models of the responses. The procedure mechanics and physics involved are explained via the surface plots. The evaluation is executed, and sufficiency is tested via the assistance of coefficient of correlation, ANOVA, and significance test (Phadke [21]). The response (wear) equation attained through CCD model is shown in Eq. 1.

Table 3 Composition ranges for 316 grade of stainless steel

Grade	C	Mn	Si	P	S	Cr	Mo	Ni	N
316	0.08	2.0	0.75	0.045	0.03	18.0	3.00	14.0	0.1

Fig. 1 Schematic diagram of experimental setup (1)air compressor, (2) flow control valve, (3) hose pipe, (4) liquid nitrogen Dewar, (5) pressure gauge, (6) flow control valve, (7) insulated stainless steel hose, (8) spindle, (9) cutting tool, (10) nozzle, (11) workpiece, (12) machine table, (13) liquid nitrogen

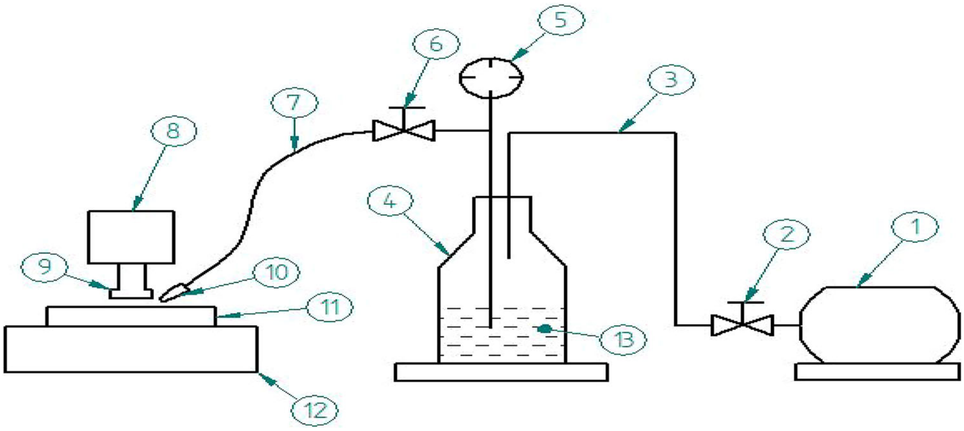

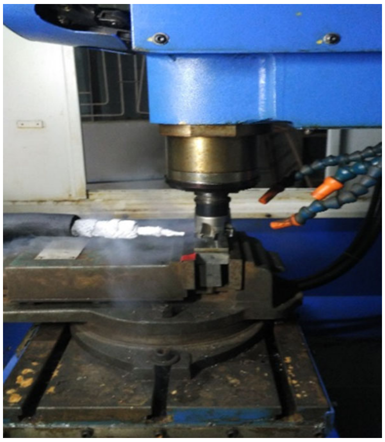

Fig. 3 Cryogenic machining region

$$\mathbf{WR} = 0.251472 + 0.0418333 * X_1 + 0.0180000 * X_2 + 0.00350000 * X_3 - 0.0520000 * X_4 - 0.0420227 * X_1 * X_1 - 0.0125227 * X_2 * X_2 + 0.00797733 * X_3 * X_3 - 0.0335227 * X_4 * X_4 + 0.00125000 * X_1 * X_2 - 0.00137500 * X_1 * X_3 - 8.75000E - 04 * X_1 * X_4 - 6.25000E - 04 * X_2 * X_3 - 0.00137500 * X_2 * X_4 - 0.00100000 * X_3 * X_4 \quad (1)$$

The coefficient term (Coef) (allude Tables 4 and 5) signifies the correlation among wear and surface roughness with the milling factors as they appear in Eqs. (1, 2). The effect of all terms (Tables 4 and 5) of wear and surface roughness is assessed by employing the significance tests. Tables 4 and 5 depict the attained "P" values (95% confidence level) of all terms via the CCD model (Ross [22]). In Table 4, for terms X_1, X_2, X_4, X_1^2, X_2^2, X_4^2 and X_1*X_2, "P" values are < 0.05 for CCD design. Henceforth, variables have substantial influence towards the wear. For the quadratic terms (i.e., X_1, X_3 and X_4), "P" values are < 0.05, signifying that the spindle speed, feed rate, and coolant type have nonlinear relation with wear. The interaction terms (X_1*X_2, X_1*X_3, X_1*X_4, X_2*X_3, X_2*X_4 and X_3*X_4) do not agree with the condition of $P < 0.05$ (more noteworthy than 0.05); therefore, these terms are considered to be insignificant terms. Significant test has been conducted for the CCD model (Mongomery [23]).

The effect of each term has been distinguished to assess the sufficiency and exactitude of the model. In the same way, as explained related to Table 4, the influence of input factors toward the response of surface roughness is depicted in Table 5 in order to identify the significance factors over the surface roughness.

Figure 5 depicts the surface plots in which two variables can be simultaneously analyzed while keeping rest of the parameters to its mid-value; these plots are acquired from the CCD to identify the behavior of wear variation with milling variables. The accompanying key perceptions have been made to comprehend the method

1. The response (wear) increases linearly with spindle speed and feed rate as represented in Fig. 5a. At higher spindle speed, the contact zone between tool and the chip interface diminishes and the tool material softens due to exposure to excess heat at the cutting edge, thus causing excess wear of the tool. Hence, at higher feed rate, more built-up edge is caused leading to more adhesion wear.
2. The response (wear) rises with an expansion of spindle speed and depth of cut as appears in Fig. 5b. Higher depth of cut causes more abrasion between contacting asperities, thus resulting in more tool wear.
3. Figure 5c highlights the ejection of LN_2 at cutting zone, reducing the sticking of workpiece material to the cutting edge and thus resulting in less built-up edge creation on tool at higher speed, and it is observed that the wear mechanism is the only abrasion.
4. From Fig. 5d, at higher feed rate more built-up edge is caused leading to fracture of tool and formation of microgroves and more adhesion wear. The higher DOC leads to more friction among the contacting asperities, subsequently softening the tool and worsening the quality of the surface.
5. Figure 5e states that at higher feed rate, by using LN_2, less adhesion wear is attained due to the formation of less built-up edge on the cutting tool edge.
6. In Fig. 5f, the significant reduction in wear rate is observed using LN_2, because chipping of cutting edge diminishes and the materials adhesion to cutting edge is less.

Tables 6 and 7 exhibit the ANOVA result of responses (wear and surface roughness) attained through the CCD model. The CCD model terms such as linear and corresponding square "P" values of CCD model are found to be less than 0.05. From Table 6, it can be noticed that the CCD model is observed to be adequate and fit for improving prediction of the response (wear).

$$Ra = 1.23988 - 0.193944 * S + 0.137611 * F + 0.0157778 * D - 0.585556 * CT - 0.0370733 * S * S - 0.0310733 * F * F + 0.0104267 * D * D + 0.191427 * CT * CT - 0.00300000 * S * F + 0.00487500 * S * D + 0.112250 * S * CT - 0.00325000 * F * D - 0.0498750 * F * CT + 0.00400000 * D * CT \quad (2)$$

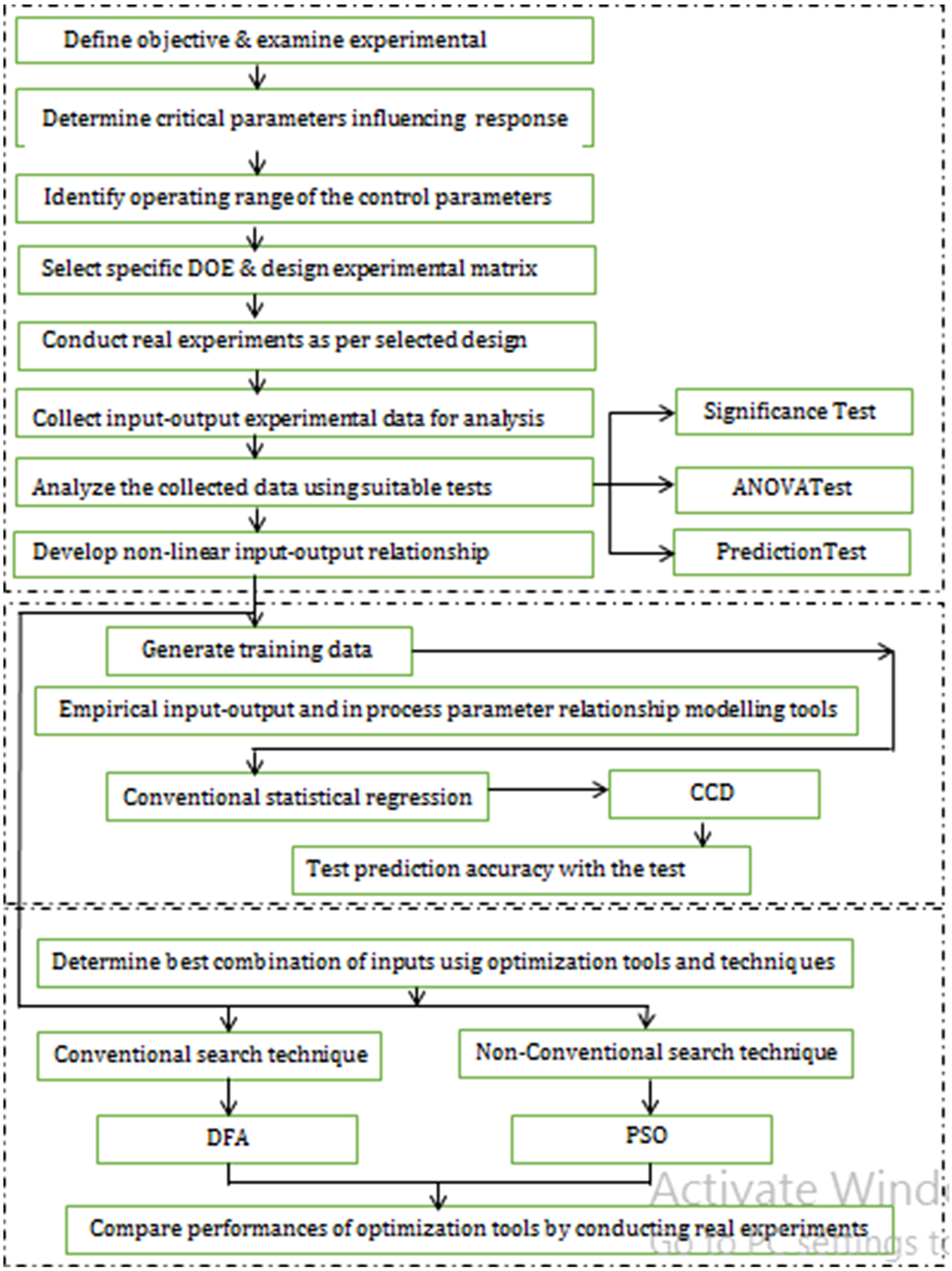

Fig. 4 Pragmatic approach for modeling and optimization: (a) modeling, (b) prediction modeling, and (c) optimization

Figure 6 depicts the surface plots in which two variables can be simultaneously analyzed while keeping rest of the parameters to its mid-value; and these plots are acquired from the CCD to identify the conduct of surface roughness variation with milling variables. The accompanying key perceptions have been made to comprehend the method

1. The response (surface roughness) value decreases linearly with spindle speed and feed rate as represented in Fig. 6a. At higher spindle speed, the chips create and split away close to the device tip with less deformation of material which thus safeguards the surface attributes of machined part causing enhanced surface quality.
2. The response decreases with an expansion of spindle speed and depth of cut as it appears in Fig. 6b. Higher depth of cut causes more abrasion between contacting asperities leading to greater surface roughness values indicating the worse surface quality.
3. Figure 6c highlights that ejection of LN_2 at cutting zone, reducing the sticking of workpiece material to the cutting edge and thus results in less built-up edge creation on tool at higher speed leading to better surface finish.

Table 4 Result of the estimated regression coefficients for response (WR) using CCD model

Term	Coef	SE Coef	T	P
Constant	0.251472	0.002302	109.240	0.000
X_1	0.041833	0.001829	22.871	0.000
X_2	0.018000	0.001829	9.841	0.000
X_3	0.003500	0.001829	1.914	0.074
X_4	− 0.052000	0.001829	− 28.430	0.000
X_1^2	− 0.042023	0.004817	− 8.724	0.000
X_2^2	− 0.012523	0.004817	− 2.600	0.019
X_3^2	0.007977	0.004817	1.656	0.117
X_4^2	− 0.033523	0.004817	− 6.959	0.000
X_1*X_2	0.001250	0.001940	0.644	0.528
X_1*X_3	− 0.001375	0.001940	− 0.709	0.489
X_1*X_4	− 0.000875	0.001940	− 0.451	0.658
X_2*X_3	− 0.000625	0.001940	− 0.322	0.752
X_2*X_4	− 0.001375	0.001940	− 0.709	0.489
X_3*X_4	− 0.001000	0.001940	− 0.515	0.613

R^2 = 99.25%, R^2 (pred) = 97.72%

4. From Fig. 6d, at higher feed rate, more built-up edge results leading to fracture of tool and formation of microgroves and more adhesion wear. The higher DOC leads to more friction among the contacting asperities, subsequently softening the tool and worsening the quality of the surface.
5. Figure 6e states that at higher feed rate, by using LN_2, less adhesion wear is attained due to the less formation of built-up edge on the cutting tool edge and leading to improved surface quality.
6. In Fig. 6f, the significant reduction in surface roughness is observed using LN_2, because chipping of cutting edge diminishes and the materials adhesion in cutting edge is less.

3.2 Evaluating the Prediction Accuracy of the CCD Model

The generated CCD model is tested for its accuracy in predicting the assistance of 15 test cases. "Appendix" indicates 15 test cases utilized on behalf of the stated purpose. The deviation (in terms of %) in the prediction of responses (wear and surface roughness) by CCD demonstrates differences in both the positive and the negative ends from the allusion zero line as shown in Figs. 7 and 8. The percent deviation for wear varies in the range between − 8.0152 and + 5.3241% for CCD method. Furthermore, the absolute deviation average (in terms of %) in prediction of CCD model is found to be 3.17%. The CCD design established regression equation is used to produce best milling conditions corresponding to required responses (Manjunath et al. [24, 25]). Similarly the percent deviation for surface roughness varies in the range between − 1.68 and + 2.68% for CCD method.

3.3 Optimization of Milling Process Parameters

The way towards deciding the superlative result among numerous probable plausible elucidations is alluded as an optimization. The CCD strategy is one of the best methods for determination of response coefficient value, as its determination coefficient points out the goodness of fit of the model (Rashmi et al. [20]). The coveted responses can be identified by incorporating the empirical model (DFA) and nontraditional (PSO) optimization strategies. In process of optimization, the attained model via CCD approach is used as objective function.

3.3.1 Traditional Method of optimization

This method uses a deterministic inquiry technique, where the result's movement will be in the unidirectional way and thus ends up in local (sub optimal) solutions. In order to decide the optimal solution, the DFA is the best way as this method is broadly used. The plots as depicted in Figs. 5 and 6 have clarified the qualitative information about the close optimal solution. Figure 9 exhibits the milling optimum values acquired via DFA method.

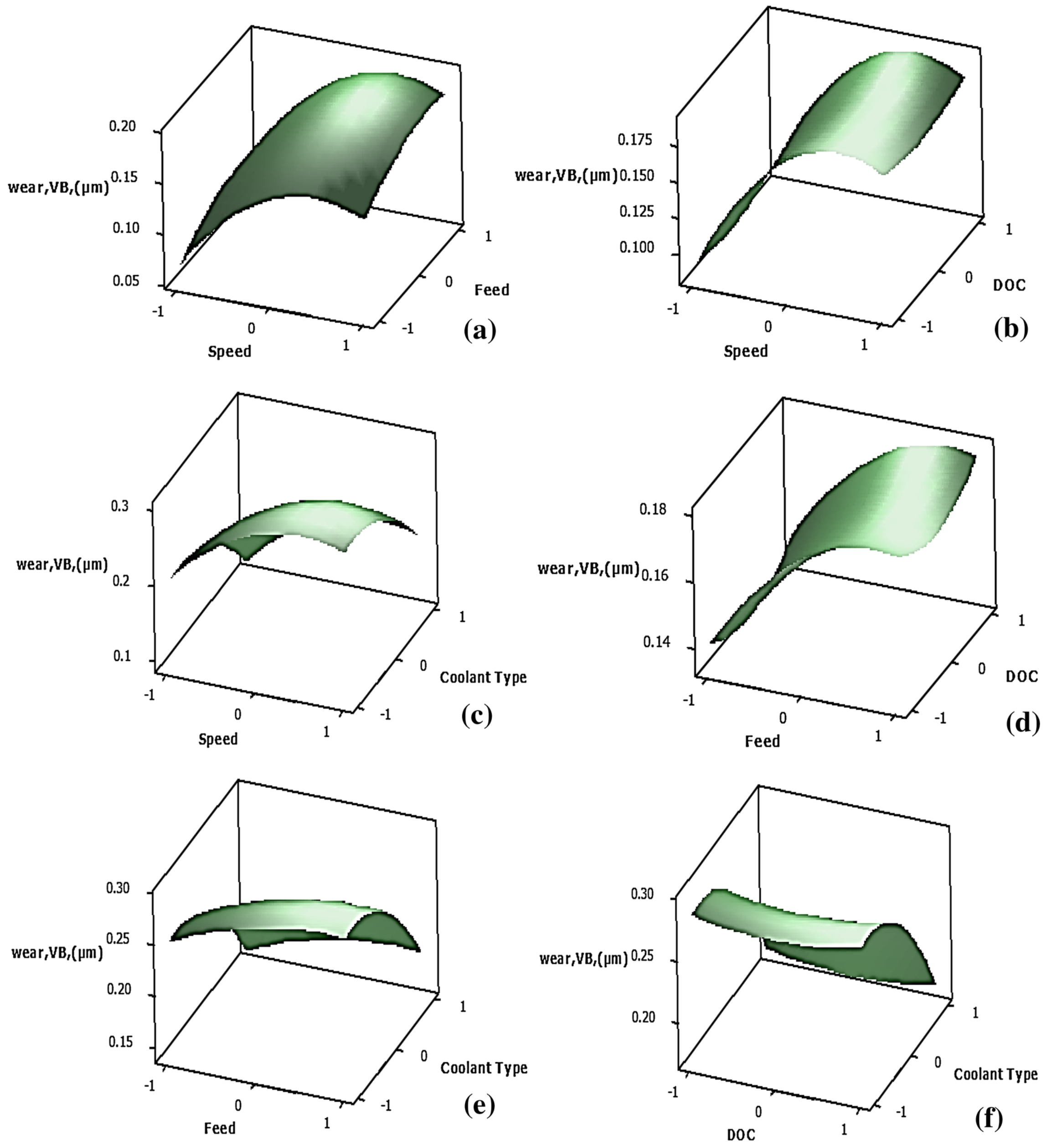

Fig. 5 Surface plots of wear rate with **a** speed and feed, **b** speed and depth of cut, **c** speed and coolant type, **d** feed and depth of cut, **e** feed and coolant type, (f) depth of cut and coolant type

3.3.2 Nonconventional Optimization

A stated optimization technique is stochastic in its way of multi-modal search and accurately simulate the close optimal outcomes (Rashmi et al. [18, 26] and Manjunath et al. [27]). The PSO enactment relies principally on the tuning of algorithm factors and the rate of convergence for the stated province. The tuning of PSO factors and the obtained outcomes are conferred in the accompanying segment.

A. PSO: PSO is established on the idea of rummaging behavior of bird flocks. PSO is prominent in manufacturing sector due to its few factors such as fine-tuning of factors, rate of convergence and simplicity of execution trademark (characteristic). In PSO, swarm is considered as a unit which is comprised of numerous distinct results and each result is considered as a particle. Every particle possesses position and velocity vector, and the search space is multidimensional. In PSO, it can be noted that each individual

Table 5 Result of the estimated regression coefficients for response (Ra) using CCD model

Term	Coef	SE Coef	*T*	*P*
Constant	1.04306	0.835303	1.249	0.230
X1	0.00003	0.000196	0.169	0.868
*X*2	0.00087	0.003966	0.218	0.830
*X*3	− 0.34717	0.391399	− 0.887	0.388
*X*4	− 0.58484	0.094950	− 6.159	0.000
*X*12	− 0.00000	0.000000	− 1.315	0.207
*X*22	0.00000	0.000004	0.162	0.874
*X*32	0.19424	0.174701	1.112	0.283
*X*42	0.15056	0.043675	3.447	0.003
*X*1***X*2	0.00000	0.000000	− 0.171	0.867
*X*1***X*3	0.00001	0.000035	0.277	0.785
*X*1***X*4	0.00011	0.000018	6.382	0.000
*X*2***X*3	− 0.00007	0.000352	− 0.185	0.856
*X*2***X*4	− 0.00050	0.000176	− 2.835	0.012
*X*3***X*4	0.00800	0.035179	0.227	0.823

R^2 = 99.67%, R^2 (pred) = 98.63%

movement of the particles takes place with a specific speed and progressively changing flight direction in view of self-flying and particle involvement in a manifold-way search domain. A systematic study is made to reach to an optimized value of PSO factors. The estimation of optimized factors of PSO is listed underneath:

Number of factors	4
Number of particles	80
Number of iterations	110
Learning rate	0.6

The PSO optimized factors return desired responses, and the corresponding milling condition is represented in Table 5. The deviations (in terms of %) vary in the range between+ 4.974% and − 4.561%. Furthermore, the PSO prediction of the absolute deviation average (in terms of %) is obtained to be 2.03%.

3.4 Confirmation Experiments

The superlative milling conditions that are the outcomes of the required responses are resolved utilizing both conventional and nonconventional optimization techniques. The goal function solely depends on the regression equation created via CCD approach. The response surface may be one-way or diverse model in gamut space. The conventional optimization strategy, DFA, involves deterministic type of explore with specific standards. The desired esteem of response (wear) achieved via DFA as appeared in Fig. 9 is observed to be more than PSO. This may conceivably be due to the multi-modal search domain. Optimum estimations of process parameters and their relating response estimates are exhibited in Table 5.

Scanning electron microscope (SEM) is ideally involved to contemplate the material surface, because of the blend of crystal clear resolution, amplification level and field profundity depth. The infinitesimal perspective of PSO optimized values appears in Table 8. Flank wear is a standout among the most vital wear to be controlled in the light of the fact that the flank confront ceaselessly and is in contacts with the machined material and eventually rises the values of cutting forces; thus, it impairs the surface (Kovacevic et al. [28] and Dhar et al. [29]). From Fig. 10, it can be noticed that the flank wear increases linearly with spindle speed under both environmental conditions, i.e., [cryogenic and wet] (Chen et al. [30]). Since the higher spindle speed produces high temperatures within very little time, additionally contact area diminishes amid the tool—workpiece interface. Thus, tool material softening leads to higher tool wear, as the cutting edge is continuously exposed to high cutting temperatures at higher spindle speed. The tool flank wear at spindle speed of 3000 rpm, feed rate of 350 mm/min and depth of cut 0.5 mm are noted as 0.24-mm and

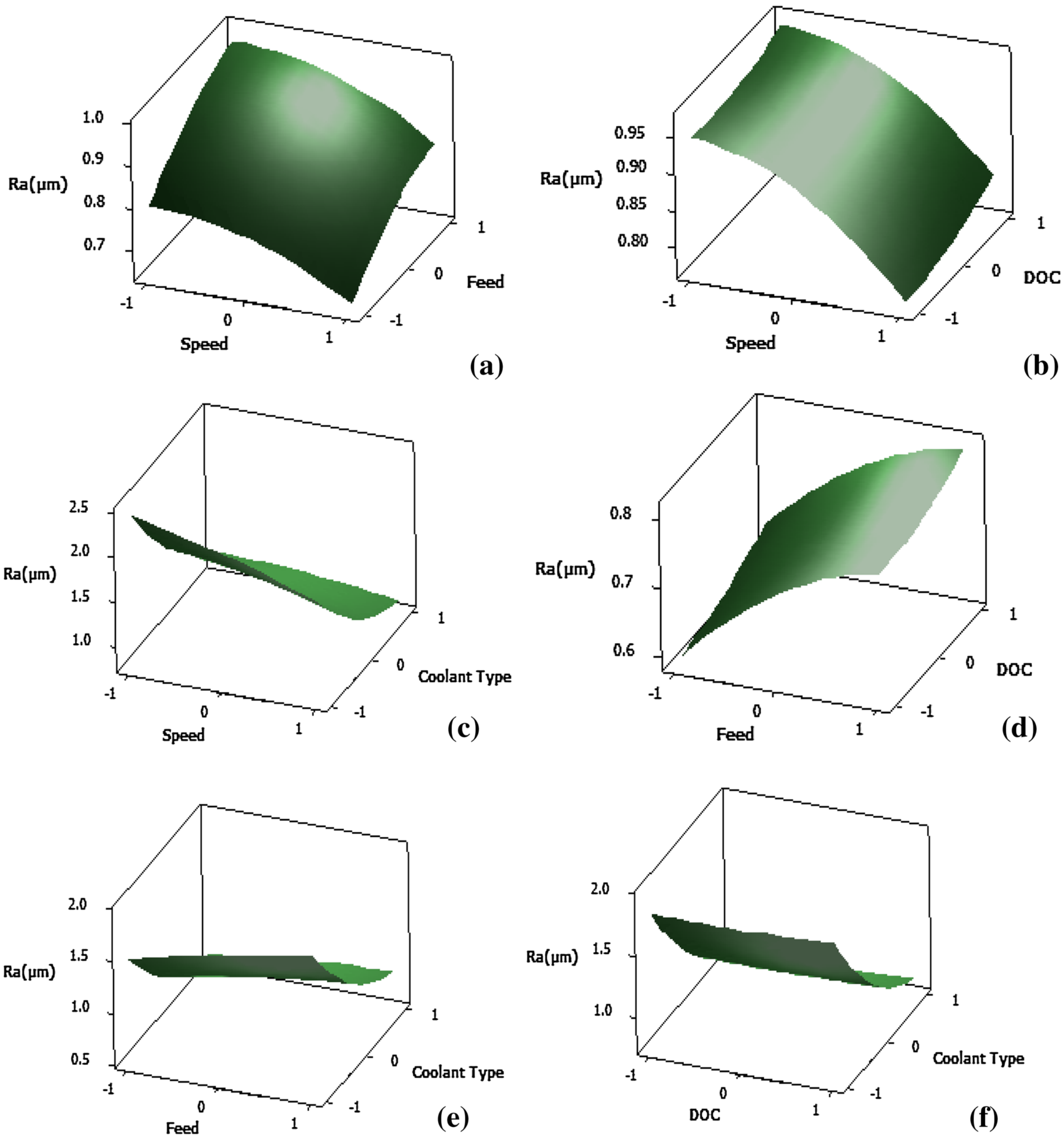

Fig. 6 Surface plots of surface roughness with **a** speed and feed, **b** speed and depth of cut, **c** speed and coolant type, **d** feed and depth of cutV, **e** feed and coolant type, **f** depth of cut and coolant type

0.142-mm under both the environmental conditions (i.e., wet, cryogenic), respectively. In LN_2 (cryogenic) condition, flank wear diminishment is observed to be 46.19% in contrast to the wet machining. The control over the mechanism of wear (i.e., abrasion and adhesion) is successful and adequate by spraying required amount of nitrogen liquid at the tool appearances of rake and flank, as the appearance of tool prompts change in wear resistance of the cutting tool and additionally decreases the cutting zone temperature [31].

Figure 10 delineates the surface and tool flank wear SEM images, by changing the speed and subsequently 3 min of machining in both the environments (i.e., wet and cryogenic). Figure 10a, b depicts the surface of the milled samples in wet and LN_2 machining methods, respectively. Figure 10a indicates that the surface quality is not that

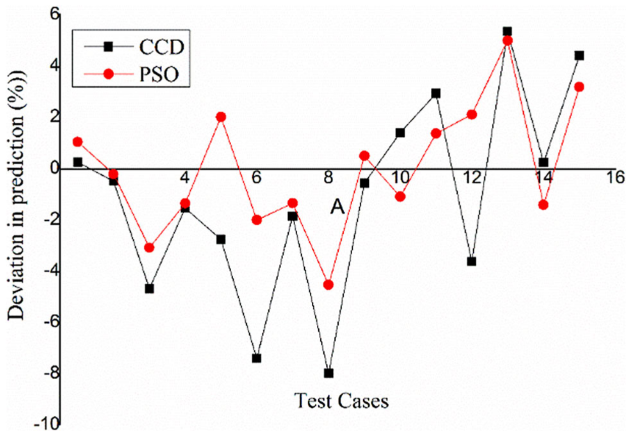

Fig. 7 Wear prediction deviation values (in terms of %) for fifteen test cases

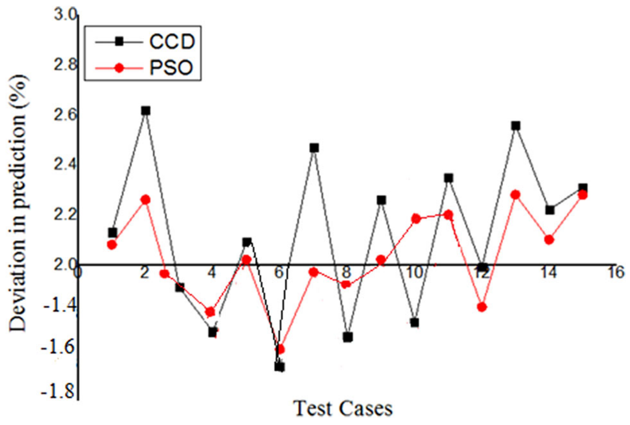

Fig. 8 Surface roughness deviation values (in terms of %) for fifteen test cases

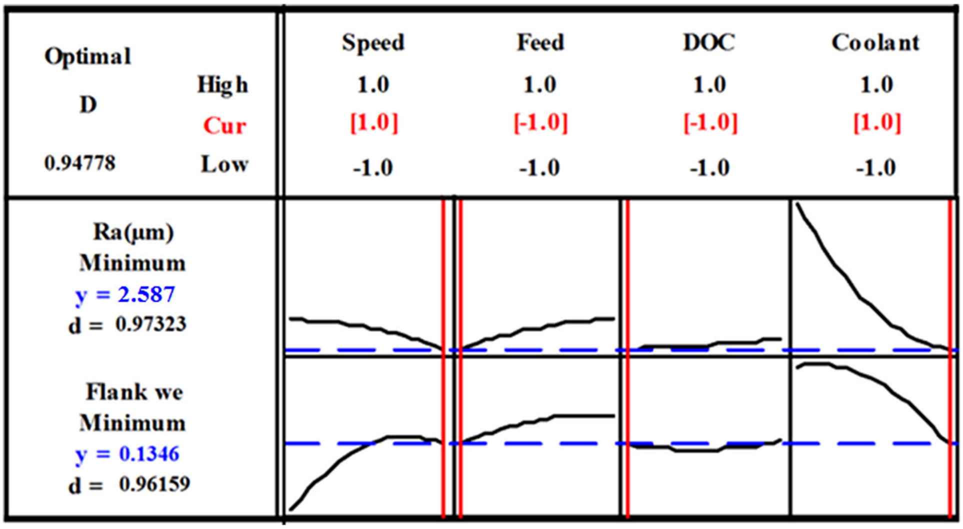

Fig. 9 Result of wear and surface roughness using DFA technique

good compared to LN_2 method as indicated in Fig. 10b. The mechanisms of wear (i.e., abrasion and adhesion) are detected on the appearance of the tool (flank face) in both conditions (i.e., wet, cryogenic). It is observed that in case of wet condition, flank wear is higher in contrast to the case of cryogenic condition. This is mainly due to the temperature increment generated in wet case of machining, leading to creation of built-up-edges (BUE) due to the robust adhesion of the chip to the tool face (rake) (Barry and Byrne [32]). Figure 10 shows that at the cutting tool, there is adhesion of machined material. Spraying of liquid nitrogen (LN2) at the zone of machining will substantially lessen the temperatures, thus reducing the adhesion and BUE formation.

The outcomes signify that even at higher spindle speed; by spraying (LN_2), tool wear can be reduced. Thus, machining with the LN_2 will enhance profitability. The consequences of PSO determine that optimized milling condition are in good concurrence with SEM images compared to CCD approach.

4 Conclusion

The experiments have been directed, and response data are gathered for various cryogenic milling conditions according to CCD outline. Both conventional and nonconventional optimization strategies have been employed to decide the optimum process factors compared to desired responses. The accompanying inferences are drawn beneath:

Table 6 Result of ANOVA test—wear

Source	DF	Seq SS	Adj SS	Adj MS	F	P
Regression	14	0.128155	0.128155	0.009154	152.01	0.000
Linear	4	0.086225	0.086225	0.021556	357.97	0.000
Square	4	0.041810	0.041810	0.010453	173.58	0.000
Interaction	6	0.000120	0.000120	0.000020	0.33	0.910
Residual error	16	0.000963	0.000963	0.000060		
Lack of Fit	10	0.000806	0.000806	0.000081	3.07	0.092
Pure error	6	0.000158	0.000158	0.000026		
Total	30	0.129119				

Table 7 Result of ANOVA test—surface roughness

Source	DF	Seq SS	Adj SS	Adj MS	*F*	*P*
Regression	14	7.47851	7.47851	0.534179	107.91	0.000
Linear	4	7.05665	0.19171	0.047927	9.68	0.000
Square	4	0.17950	0.17950	0.044875	9.07	0.001
Interaction	6	0.24235	0.24235	0.040392	8.16	0.000
Residual error	16	0.07921	0.07921	0.004950		
Lack of fit	10	0.07611	0.07611	0.007611	14.76	0.002
Pure error	6	0.00309	0.00309	0.000516		
Total	30	7.55771				

Table 8 Summarizes the optimal process parameters

Optimization tool	Milling condition					
	Spindle speed	Feed rate	Depth of cut	Coolant	Wear	Ra
DFA	3000	350	0.5	1	0.134	2.587
PSO	3000	350	0.5	1	0.139	2.629
Experimental	3000	350	0.5	1	0.142	2.643

- It is to be noticed that CCD model is observed to be statistically sufficiently great in response prediction. In order to carry out and attain the optimized values, the achieved response (regression equation) is used as a goal (objective) function.
- The desired responses (wear and surface roughness) and the relative milling prerequisite are resolved using traditional and nontraditional methods. It is fascinating to take a note that nontraditional (PSO) strategy outperforms DFA in determining the best milling condition that generate required responses.
- From the attained results, it can be derived that PSO produces the closure values as compared to the desirability approach.
- The wear and surface roughness prediction deviation values (in terms of %) for fifteen test cases indicate that the deviations attained through PSO are better as compared to the CCD technique.
- SEM micrographs and the surface plots indicate that usage of LN_2 and spray of LN_2 at the contact asperities will yield better surface roughness and diminishes the tool wear as compared to the flood coolant during machining.

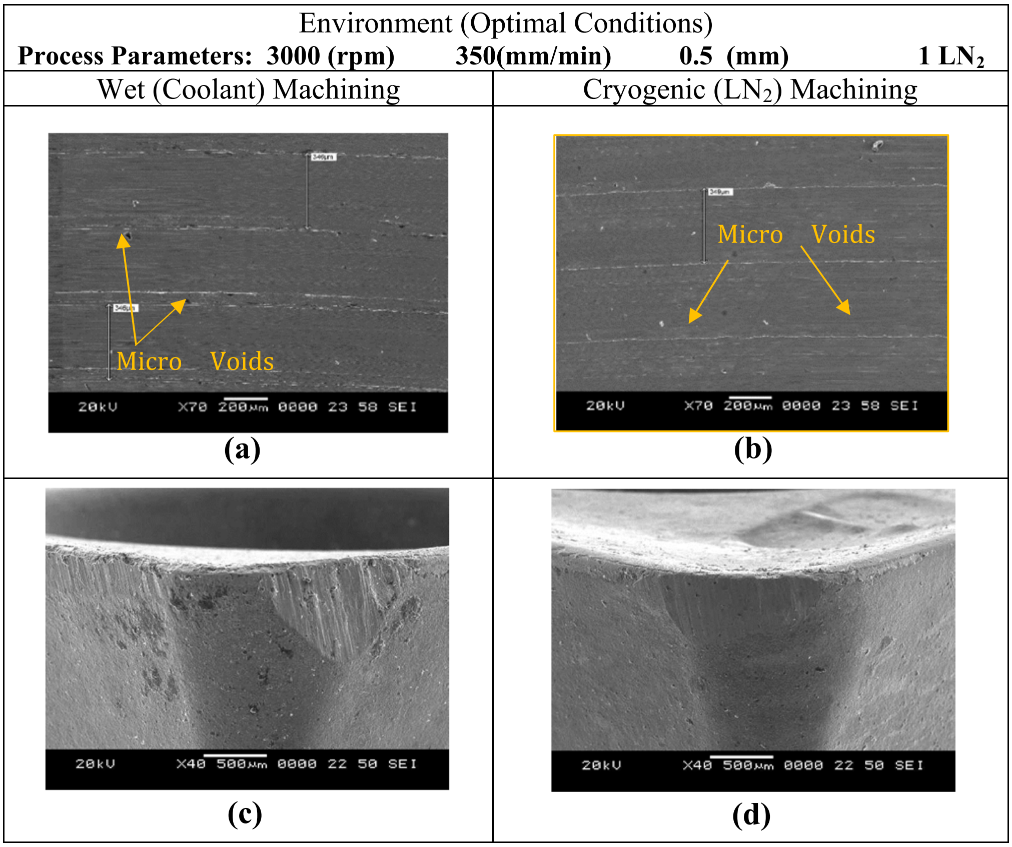

Fig. 10 Microscopic view of milling samples (**a**–**b**) surface roughness and (**c**–**d**) tool flank wear at different machining condition

- It is substantiated experimentally that LN_2 machining enhances the milling performance of SS316 at higher spindle speed by aiding the lower flank wear in contrast to the wet machining. Thus, LN_2 machining escalates the profitability with greater quality. Thus, LN_2 machining is a viable key for machining at higher speed.

Acknowledgements I would like to thank NITK, Surathkal, for providing facilities to carry out my research work.

Appendix: Test Cases

SL. no.	Process variables				Experimental	
	S	F	D	CT	Wear rate	Surface roughness
1	2100	465	1.1	− 1	2.27	2.13
2	2800	379	0.7	0	2.41	2.62
3	1700	364	0.9	1	1.93	1.91
4	1200	420	1.3	1	1.74	1.73
5	2000	457	0.8	0	2.24	2.09

Appendix continued

SL. no.	Process variables				Experimental	
	S	F	D	CT	Wear rate	Surface roughness
6	1500	500	1.2	1	1.99	1.83
7	2600	440	0.7	1	1.13	2.47
8	1285	520	1.3	1	0.62	1.71
9	2300	500	0.8	0	2.13	2.26
10	1392	390	0.9	1	1.66	1.77
11	2500	485	1	− 1	2.32	2.35
12	1850	510	1.4	1	2.01	1.99
13	2720	400	1.1	− 1	2.66	2.56
14	2200	380	0.8	0	2.17	2.22
15	2450	510	0.6	− 1	2.38	2.31

References

1. Cambri B M, *J Mat Processing Technology* **56** (1996) 786.
2. Shaw M C, Pigott J D, and Richardson L P, *Am Soc. Mech. Eng.* **71** (1951) 45.
3. Cassin C, and Boothroyd G, *J Mech Eng Sci* **7** (1965) 67.
4. Baradie M A, *J Mater Process Technol* **56** (1996b) 798.

5. Pusavec F, Kramar D, Krajnik P, and Kopac J, *J Cleaner Prod* **18** (2010) 1211.
6. Hong S Y, and Broomer M, *Clean Prod Process* **2** (2000) 157.
7. Hong S Y, Ding Y, and Jeong J, *Mach Sci Technol* **6** (2002) 235.
8. Bordin A, Bruschi S, Ghiotti A, and Bariani P F, *Wear* **328** (2015) 89.
9. Jerold B D, and Kumar M P, *Cryogenics* **52** (2012) 569.
10. Umbrello D, *J Adv Manuf Technol* **64** (2015) 633.
11. Umbrello D, *Int J Adv Manuf Technol* **54** (2011) 887.
12. Klocke F, Settineri L, Lung D, Priarone PC, and Arft M, *Wear* **302** (2013) 1136.
13. Tandon V, Mounayri H E, and Kishawy H, *Int J Mach Tools Manuf* **42** (2002) 595.
14. Basker N, Asokan P, Saravanna R, and Probhaharan G, *Int J Adv Manuf Technol* **25** (2005) 10781088.
15. Mukherjee I, and Kumar R P, *Comput Ind Eng* **50** (2006) 15.
16. Raja S B, and Baskar N, *Expert Syst Appl* **39** (2012) 5982.
17. Julie Z, Joseph C, and Daniel K, *J Mater Process Technol* **184** (2007) 233.
18. Rashmi L M, Karthik Rao M C, Arun Kumar S, Shrikantha S Rao, and D'Souza R J, *J Braz Soc Mech Sci Eng* **39** (2016) 3541.
19. Reddy S K, and Rao P V, *Int J Adv Manuf Technol* **28** (2006) 463.
20. Rashmi L M, Karthik R M C, Arun Kumar S, Shrikantha S R, D'Souza R J, *Mater Manuf Process* **33** (2017) 1406.
21. Phadke M S, *Quality engineering using robust design*, Prentice Hall, New Jersey (1989).
22. Ross P J, *Taguchi techniques for quality engineering*, McGraw-Hill, New York (1996).
23. Montgomery D C, *Design and analysis of experiments*, Wiley, New York (2008).
24. Manjunath P, Krishna P, and Parappagoudar B, *Int J Adv Technol* (2016), http://dx.doi.org/10.1007/s00170-016-8416-8.
25. Manjunath P, Krishna P, and Parappagoudar B, *Aust J Mech Eng* (2015) http://dx.doi.org/10.1080/14484846.2015.1093231.
26. Rashmi L M, Karthik R M C, Arun Kumar S, Shrikantha S R, and Mervin A H, *Int J Precis Eng Manuf* **19** (2018) 695.
27. Manjunath P, Arun Kumar S, and Parappagoudar B, *J Manuf Process* **32** (2018) 199.
28. Kovacevic R, Cherukuthota C, and Mzurkiewiez M, *Int J Mach Tools Manuf* **35** (1995) 1459.
29. Dhar N R, Paul S, and Chattopadhyay A B, *Wear* **249** (2002b) 932.
30. Chen Z, Atmadi A, Stephennon D A, and Liang S Y, *Ann CIRP* **49** (2000) 53.
31. Yakup Y, and Muammer N, *Int J Mach Tool Manuf* **48** (2008) 947.
32. Barry J, and Byrne G, *Ann CIRP* **51** (2002) 65.

View publication stats
