# Supplementary material for: Dataset on flank wear, cutting force and cutting temperature assessment of austenitic stainless steel AISI316 under dry, wet and cryogenic during face milling operation
Source: Data Brief. 2019 Aug 26;26:104389. doi: 10.1016/j.dib.2019.104389 (PMC6737182; doi:10.1016/j.dib.2019.104389)
Supplement: Multimedia component 2 [file mmc2.zip › dib_104389_mmc2.PDF]

Journal of Materials Science & Surface Engineering, 5(6): 653-656
ISSN (Online): 2348-8956; 10.jmsse/2348-8956/5-6.3

Machinability Study of Austenitic Stainless Steel under Wet and Cryogenic Treatment in Face Milling

Karthik Rao M C[1] . Arun Kumar Shettigar[1] . Rashmi L Malghan[2] . Shrikantha S Rao[2] . Mervin A Herbert[2]

[1]Manipal Institute of Technology, Department of Mechatronics Engineering, Manipal, India.
[2]National Institute of Technology Karnataka, Department of Mechanical Engineering, Surathkal, India.

ABSTRACT
In manufacturing industries, the rise in cutting temperature during machining operations influences the surface finish of the product. Since in metal cutting the usage of coolant is less efficient compared to cryogenic machining in reducing the rise in temperature in tool-workpiece interface. The paper shows the impact of cryogenic fluid nitrogen (LN_2 at -196 ^{0}C) on surface integrity and cutting temperature in processing of AISI 316 and the outcomes were contrasted over the wet machining. The outcomes have revealed that cryogenic machining has yielded better surface finish when contrasted with traditional wet machining.

© 2017 JMSSE and Science IN. All rights reserved
NCMDMM-2017, August 3-5, 2017, Karnataka, India.

ARTICLE HISTORY
Received 15-07-2017
Revised 25-07-2017
Accepted 29-07-2017
Published 28-08-2017

KEYWORDS
Cryogenic machining, Wet machining, Face Milling, One parametric approach, Cutting Temperature, surface roughness

Introduction

Austenitic stainless steel is a standout amongst the most engineering materials with a wide assortment of utilizations. Better resistance than erosion and similarity in high temperature and high vacuum has especially settled on it an appealing decision. Stainless steel is otherwise called corrosion resistant steel, since it is an iron-based steel combination, which contains least 11% chromium. Chromium exhibit in it keeps it from getting corrode. At the point when conventional carbon steel is presented to rain water, it erodes effectively because of the development of a dark coloured iron oxide at first glance, which is regularly called as rust. In any case, when more than around 10% chromium is added to standard steel, the oxide at first glance is changed. Stainless Steel for the most part has high malleability and weldability properties.

Machining industries are utilizing ordinary liquid cooling at the metal cutting area to defeat the temperature raises [1]. Nonetheless, it was discovered that flood cooling strategy not bunch to diminish the cutting temperature at device chip interface at higher cutting liquids [2,3]and regular cutting liquids is concoction contaminants causes a few health, environmental issues and extra transfer cost [4]. Likewise, it is determined that the related costs identified with cutting liquids are 30% of aggregate assembling costs [5]. Albeit a few ecologically cognizant controls limited the enterprises as far as utilization and transfer of customary slicing liquids because of wellbeing risky and natural contamination affect [6]. In this way, there is a need to focus on green assembling to defeat natural contamination, cutting liquid transfer, support, upkeep and lower profitability issues. Cryogenic machining is one of the alternative approaches. Subsequently, in this study cryogenic splash cooling system is utilized, i.e. streaming of the Liquid nitrogen (LN2) at – 196°C at the tool-workpiece interface. LN2 generously decreases the temperatures at thetool-chip interface and creates much lower friction coefficient [7]. A. Bordin et al. illustratedlow tool wear and low surface roughness in cryogenic machining contrasted with dry machining amid machining of titanium amalgam [8]. B.D. Jerold et al. demonstrated that CO_2 yielded better instrument wear contrasted with the wet machining for AISI 1045 steel [9]. D. Umbrelloillustrated better surface finish in cryogenic machining contrasted with the dry machining amid machining of AISI 52100 steel [10,11]. F. Klocke et al. observed lower tool wear and reduced surface roughness in cryogenic condition compared to minimum quantity lubrication (MQL) machining conditions because of the considerable decrease of the cutting temperatures [12]. Thus, cryogenic machining expands the efficiency and quality of the item in the machining of gamma titanium aluminides contrasted with the other wet, MQL machining. S. Y. Hong et al. completed monetary and natural investigation in the turning of AISI 304 and inferred that cryogenic approach is more beneficial and help to achieve more prominent profitability contrasted with emulsion cooling, without wellbeing and ecological concerns [6].

To the author's knowledge very less report is accessible on machining of AISI 316 under cryogenic condition. From the literature, it can be observed that cryogenic cooling increases the tool life, attains better surface finish with amid machining of hard to cut material, regardless of many points of interest it over cools the surface, increases the hardness of the machined surface. Thus, the point of the current investigation is to consider the impact of LN_2 as the coolant on cutting temperature and surface integrity in the processing of AISI 316 over the wet machining.

Experimental

Materials and Methods

Table 1 depicts the experimental conditions utilized in the current work. CNC Spark DTC-12 was utilized to carry out

Corresponding Author: Karthik Rao, Tel: +91 9986087148
Email: rao.karthik@manipal.edu
© 2017 Science IN. All rights reserved

Face milling experiments on AISI 316 stainless steel. LN_2cryogenicmachining setup is delineated in Figure 1 and metal cutting region is shown in Figure 2. Experiments were carried out in650, 750 and 850 rpm, constant feed rate of 250 mm/min and depth of cut 0.8 mm. The boundary limit of parameters depended on the preliminary experiments conducted.

Table 1: Experimental conditions

Workpiece Material & Size	AISI 316 (100mm x 40mm x 30)
Face Milling Process Parameters	Spindle Speed: 650,700,750,800,850 rpm<br>Feed Rate: 250 mm/min<br>Depth of Cut: 0.8mm
Environments	Wet (Flood Coolant).<br>Cryogenic (LN_2)

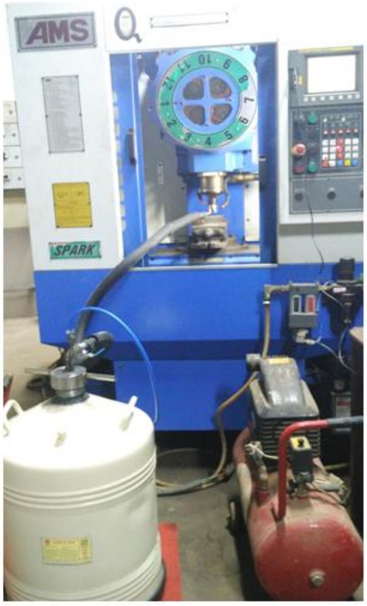

Figure 1: Cryogenic machining setup

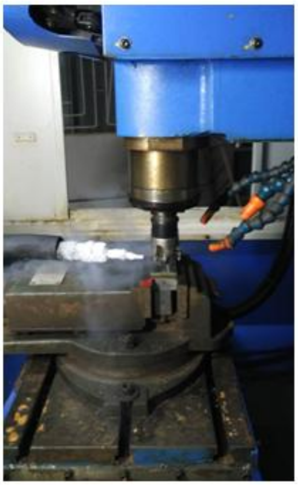

Figure 2: Cryogenic machining region

Cryogenic Cooling Method

A minimal cost setup of cryogenic cooling was utilized to encroach the LN_2 at the tool-workpiece interface. The setup includes TA55 cryocan, it is utilized to stock up the LN_2 via the air compressor. Later on, the pressurized LN_2 is provided through the spout to the tool-workpiece interface with the pressure of 3 bar and flow rate of 0.36 l/min.

Measurement of Performance Characteristics

The surface roughness of each cryogenically machined surface was measured using the Mitutoyo surface roughness analyser. Three readings at different locations were measured on each machined surface and the average of it was considered as the conclusive estimation of surface roughness estimation. Infrared thermometer was utilized to quantify the cutting temperatures at the tool-workpiece interface.

Results and Discussion

Effect of cryogenic LN2 on cutting temperature

From the Figure 3, it is observed that the cutting temperature increases linearly with the spindle speed in both cryogenic and wet machining. The prominent cutting temperatures at a spindle speed of 650rpm and feed rate of 250 mm/min was 112.8 ˚C for wet and 43.6˚C for cryogenic machining. It is noticed that 69.4% of cutting temperature decreased by using LN_2over wet conditions. This is because of the low temperature of LN_2 which was influenced at the tool-workpiece interface as it retains the cutting temperatures at the cutting region.

The cutting temperatures raised from 43.6˚C to 103.5˚C and 112.8˚C to 335.9˚C in cryogenic and wet machining respectively, when the spindle speed raised from 650 to 850 rpm at a constant feed rate of 250 mm/min. In the study using LN2, reduced the cutting temperature from 69.2% to 81.4% over the wet machining. From the Figure 3 it can be seen that, the rise in temperature diminished for cryogenic condition compared to wet machining. It is also observed that there is a decrement in the cryogenic effect with increase in spindle speed this is because of the accumulation of chips at tool face, so it hinders the entrance of LN_2 in-turn reducing the cryogenic effect [13].

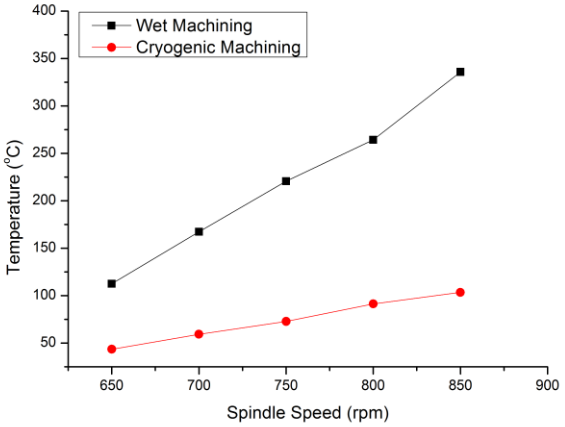

Figure 3: Cutting Temperature Variation

Effect of cryogenic LN2 on surface finish

The surface roughness estimation of machined surface of various spindle speed under the cryogenic and wet machining conditions is shown in Figure 4 and observed that surface roughness diminishes with increment in spindle speed. This effect is because of increment of cutting temperatures with an increase in spindle speed, as this

© 2017 Science IN. All rights reserved

causes to thermal softening of workpiece material and more expulsion of surface discontinuities and blemishes at the higher spindle speed resulting in a better surface finish. The surface roughness value is 0.84μm and 1.96 μm for cryogenic and wet machining respectively at the spindle speed of 650 rpm, feed rate of 250 mm/min and depth of cut of 0.8 mm. By using LN_2, 37.62% of surface finish was enhanced over the wet machining. This is because of the concurrent use of LN_2 at the tool-workpiece interface lessens the cutting temperatures in the metal cutting region, thus leading to less adhesion at the tool-workpiece interface and creating less tool mark on the machined surface, in this manner better surface finish was accomplished.

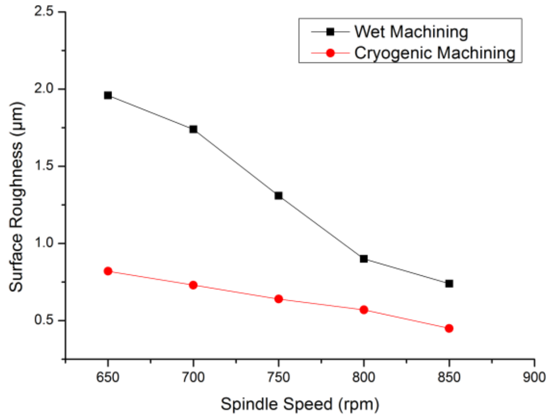

Figure 4: Surface Roughness (Ra) Variation

Spindle Speed (rpm)	Environment	
	Wet Machining	Cryogenic Machining
Low speed 650 (rpm)	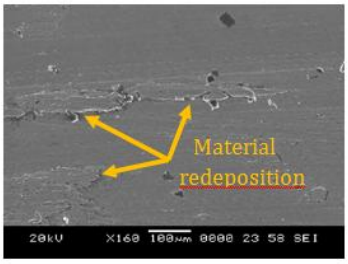	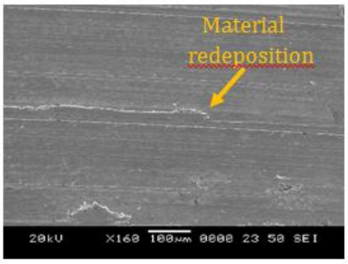
High speed 850 (rpm)	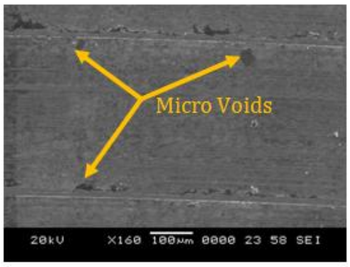	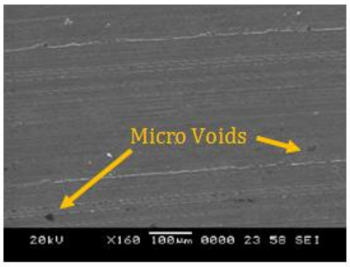

Figure 5: Machined surface SEM images at different environment

Spindle Speed (rpm)	Environment	
	Wet Machining	Cryogenic Machining
Low speed 650 (rpm)	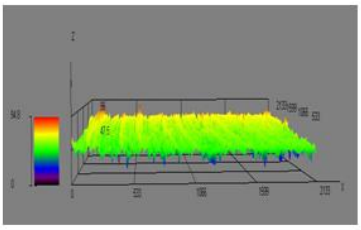	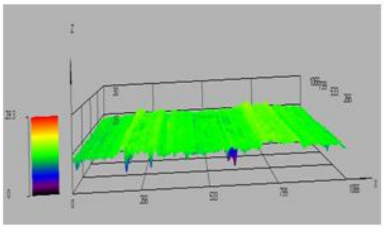
High speed 850 (rpm)	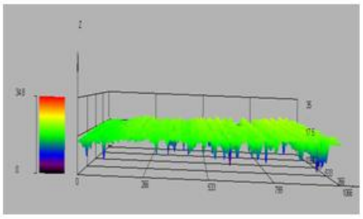	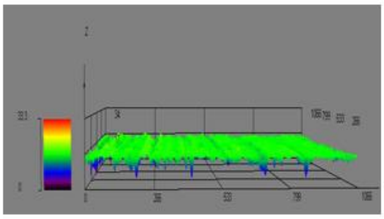

Figure 6: Machined surface 3D confocal images at different environment

© 2017 Science IN. All rights reserved

From the Fig. 5 and Fig. 6, it can be observed that micro voids, less depositions of material and surface peaks of lower intensity have been located in cryogenic machining. The surface roughness value reduction in cryogenic machining was observed to be 39.18 % to 58.16 % compared to wet machining at varying spindle speed from 650 rpm to 850 m/min, constant feed rate of 250 mm/min and constant depth of cut of 0.8 mm.

Conclusions

The major conclusions that can be drawn from the experimental study are:

1. LN2 viably controlled the metal cutting region temperature, thus facilitated better surface finish over wet machining.
2. By using cryogenic conditions 69.4% of the cutting temperature was reduced over wet machining.
3. Experimentally it was substantiated that cryogenic machining essentially enhanced the performance of face milling even at higher spindle speed during machining of AISI 316. In this manner, cryogenic machining expands the efficiency with better quality.

Acknowledgements

The authors would like to sincerely thank the Department of Mechanical Engineering National Institute of Technology Karnataka, Surathkal and Manipal Institute of Technology, Manipal,for providing research facilities.

References

1. M.A. Cambri Baradie, Cutting fluids: Part I. Characterisation. J Mat. Processing Technology, 1996a, 56 (1), 786–797.
2. M.C. Shaw, J.D. Pigott and L.P. Richardson, L.P, The effect of cutting fluid upon chip-tool interface temperature. Trans. ASME,1951, 71, 45–56.
3. C. Cassin and G. Boothroyd, Lubrication action of cutting fluids. J Mechanical Engineering Science, 1965, 7(1).67–81.
4. M.A Baradie, Cutting fluids: Part II. Recycling and clean machining. J Mat. Processing Technology, 1996b, 56 (1), 798–806.
5. F. Pusavec, D. Kramar, P. Krajnikand J. Kopac, Transitioning to sustainable production – Part II: Evaluation of sustainable machining technologies.J Cleaner Production, 2010, 18(12), 1211–1221
6. S.Y. Hong and M. Broomer, Economical and ecological cryogenic machining of AISI 304 austenitic stainless steel. Clean Products and Processes, 2000, 2(3),157–166.
7. S.Y. Hong, Y. Ding and J. Jeong, Experimental evaluation of friction coefficient and liquid nitrogen lubrication effect in cryogenic machining. Machining Science and Technology, 2001, 6(2), 235–250.
8. A. Bordin, S. Bruschi, A. Ghiotti and P.F. Bariani, Analysis of tool wear in cryogenic machining of additive manufactured Ti6Al4V alloy. Wear, 2015, 328, 89–99.
9. B.D. Jerold and M.P. Kumar, Experimental investigation of carbon-dioxide and liquid nitrogen cryogenic coolants turning of AISI 1045 steel. Cryogenics, 52(10), 2012, 569-574.
10. D. Umbrello, Analysis of the white layers formed during machining of hardened AISI 52100 steel under dry and cryogenic cooling conditions. J AdvManufTechnol, 2015, 64(5–8), 633–642
11. D. Umbrello, Influence of material microstructure changes on surface integrity in hard machining of AISI 52100 steel. Int J AdvManufTechnol, 2011, 54(9–12), 887–898.
12. F. Klocke, L. Settineri, D. Lung, P.C. Priarone and M. Arft, High performance cutting of gamma titanium aluminides: Influence of lubricoolant strategy on tool wear and surface integrity. Wear, 2013, 302(1),1136–1144.
13. Y.Yildiz, and M. Nalbant, A review of cryogenic cooling in machining processes. International Journal of Machine Tools and Manufacture, 2008, 48 (9), 947–964.

© 2017 Science IN. All rights reserved
